# Supplementary material for: The Ecology of Unsheltered Homelessness: Environmental and Social-Network Predictors of Well-Being among an Unsheltered Homeless Population
Source: Int J Environ Res Public Health. 2021 Jul 8;18(14):7328. doi: 10.3390/ijerph18147328 (PMC8306744; doi:10.3390/ijerph18147328)
Supplement: Supplementary file 1 [file ijerph-18-07328-s001.zip › ijerph-1266463-supplementary.pdf]

Supplementary Table S1. SF-36 questions used to calculate scores for general health and emotional well-being scales from the SF-36

SF-36 Item	General Health Scale					
1.	In general, would you say your health is:	(1) Excellent	(2) Very Good	(3) Good	(4) Fair	(5) Poor
		**Definitely True**	**Mostly True**	**Don't Know**	**Mostly False**	**Definitely False**
33.	I seem to get sick a little easier than other people.	(1)	(2)	(3)	(4)	(5)
34.	I am as healthy as anybody I know.	(1)	(2)	(3)	(4)	(5)
35.	I expect my health to get worse.	(1)	(2)	(3)	(4)	(5)
36.	My health is excellent.	(1)	(2)	(3)	(4)	(5)

SF-36 Item	Emotional Well-Being Scale						
	How much of the time during the past 4 weeks…	**All of the time**	**Most of the time**	**A good bit of the time**	**Some of the time**	**A little of the time**	**None of the time**
24.	Have you been a very nervous person?	(1)	(2)	(3)	(4)	(5)	(6)
25.	Have you felt so down in the dumps that nothing could cheer you up?	(1)	(2)	(3)	(4)	(5)	(6)
26.	Have you felt calm and peaceful?	(1)	(2)	(3)	(4)	(5)	(6)
28.	Have you felt downhearted and blue?	(1)	(2)	(3)	(4)	(5)	(6)
30.	Have you been a happy person?	(1)	(2)	(3)	(4)	(5)	(6)

Supplementary Table S2. Univariate linear regression models examining associations between sociodemographic characteristics, substance use, and health conditions on general health and emotional well-being.

	General Health		Emotional Well-Being	
	β	95% CI	β	95% CI
**Sociodemographic Characteristics**				
Age	0.1	(-0.22, 0.45)	0.2	(-0.04, 0.54)
Gender				
Female (reference)	---	---	---	---
Male	**13.9*** **	**(6.56, 21.2)**	**6.9***	**(0.5, 13.4)**
Non-Binary	9.0	(-17.8, 35.8)	-2.1	(-25.6, 21.6)
LGBTQI+				
No (reference)	---	---	---	---
Yes	-10.5	(-21.7, 0.79)	-2.2	(-11.9, 7.6)
Ethnicity				
Non-White (reference)	---	---	---	---
White	-6.6	(-13.7, 0.45)	-3.5	(-9.6, 2.6)
Highest Level of Education				
K-11th Grade (reference)	---	---	---	---
GED or High School	**8.3***	**(0.43, 16.2)**	5.5	(-1.3, 12.4)
Trade School or Any Higher Education	8.2	(-0.69, 17.2)	3.4	(-4.3, 11.2)
Veteran				
No (reference)	---	---	---	---
Yes	-1.8	(-12.9, 10.6)	2.6	(-7.6, 12.7)
Has Caseworker				
Yes (reference)	---	---	---	---
No	6.4	(-1.6, 14.6)	3.5	(-3.4, 10.5)
Lifetime Homelessness Duration				
1 year or less (reference)	---	---	---	---
1 year – 5 years	-5.2	(-14.9, 4.5)	-2.1	(-10.4, 6.2)
5 years – 10 years	0.4	(-10.0, 10.7)	5.0	(-3.9, 13.9)
10 years +	-4.9	(-15.7, 5.9)	-1.9	(-11.2, 7.4)
Sleeps in Encampment with Other PEH				
No (reference)	---	---	---	---
Yes	3.6	(-3.4, 10.6)	-1.5	(-7.6, 4.6)
**Substance Use**				
Alcohol Abuse				
No (reference)	---	---	---	---
Yes	-6.6	(-13.5, 0.2)	**-11.8*** **	**(-17.5, -6.0)**
Drug Abuse				
No (reference)	---	---	---	---
Yes	-5.2	(-12.1, 1.8)	**-6.3***	**(-12.2, -0.34)**
**Health Conditions**				
Number of Chronic Conditions (*Excluding Mental Health*)	**-7.1*** **	**(-8.4, -5.9)**	**-2.4*** **	**(-3.7, -1.1)**
Mental Health Diagnosis				
No (reference)	---	---	---	---
Yes	**-14.2*** **	**(-20.9, -7.5)**	**-17.6*** **	**(-23.2, 12.1)**

Significance values: *p ≤ 0.05, **p ≤ 0.01, ***p ≤0.001

Supplementary Table S3. Univariate linear regression models examining associations between biophysical environment and social support networks on general health and emotional well-being.

	General Health		Emotional Well-Being	
	β	95% CI	β	95% CI
**Biophysical Environment**				
Season				
Summer (reference)	---	---	---	---
Fall	-4.2	(-13.7, 5.3)	-3.6	(-12.0, 4.8)
Winter	**-13.9****	**(-23.6, -4.2)**	-3.0	(-15.0, 3.3)
Spring	-4.5	(-14.9, 5.9)	-5.9	(-11.4, 5.4)
Daily Minimum Temperature (°C)	0.4	(-0.02, 0.7)	0.1	(-0.2, 0.4)
Daily Maximum Temperature (°C)	**0.4***	**(0.02, 0.8)**	0.1	(-0.3, 0.4)
Precipitation				
No (reference)	---	---	---	---
Yes	1.0	(-6.6, 8.6)	4.9	(-1.7, 11.4)
Number Nights Spent Indoors in Past Week	**1.7***	**(0.14, 3.2)**	0.6	(-0.7, 1.9)
**Social Network Characteristics**				
Network Size	-0.7	(-2.0, 0.5)	0.7	(-0.4, 1.8)
Number of Family in Network	-1.3	(-3.5, 0.5)	1.1	(-0.5, 2.6)
Number of Friends in Network	-0.5	(-2.3, 1.2)	0.9	(-0.6, 2.4)
Number of Emotional Supports in Network	-0.8	(-2.1, 0.5)	1.0	(-0.2, 2.1)
Number of Material Supports in Network	-1.0	(-2.4, 0.3)	0.8	(-0.4, 1.9)
Number of Financial Supports in Network	-0.8	(-2.1, 0.6)	**1.3***	**(0.1, 2.5)**
Number of Network Members With Whom Participant Uses Alcohol	-1.1	(-2.8, 0.7)	0.3	(-1.2, 1.8)
Number of Network Members With Whom Participant Uses Drugs	-1.7	(-4.3, 0.8)	-1.8	(-3.9, 0.3)
Number of Trusted Network Members	-0.6	(-1.9, 0.7)	**1.5***	**(0.4, 2.6)**
Number of Network Members Who Upset Participant in Past 30 Days	**-3.0***	**(-5.4, -0.5)**	**-4.1*****	**(-6.2, 1.9)**
Number of Housed Network Members	0.77	(-1.6, 1.5)	**1.3***	**(0.01, 2.6)**
Number of Unhoused Network Members	-1.6	(-3.6, 0.4)	-0.2	(-1.9, 1.5)

Significance values: *p ≤ 0.05, **p ≤ 0.01, ***p ≤0.001

Supplementary Table S4. Independent samples two-tailed t-test results comparing social network composition differences between men and women among N=245 unsheltered PEH in Nashville, TN.

	Men n = 167		Women n = 74		t-value
	**Mean**	**SD**	**Mean**	**SD**	
**Social Network Characteristics**					
Network Size	4.8	2.7	5.3	2.7	-1.2
Number of Family in Network	1.9	1.8	2.3	2.1	-1.4
Number of Friends in Network	2.5	1.9	2.7	1.9	-0.8
Number of Emotional Supports in Network	4.0	2.6	4.5	2.8	-1.2
Number of Material Supports in Network	4.0	2.6	4.5	2.6	-1.2
Number of Financial Supports in Network	3.3	2.6	3.7	2.3	-1.3
Number of Network Members With Whom Participant Uses Alcohol	1.7	2.1	1.1	1.5	**2.4***
Number of Network Members With Whom Participant Uses Drugs	0.9	1.5	0.7	1.0	1.5
Number of Trusted Network Members	4.3	2.6	4.4	2.8	-0.2
Number of Network Members Who Upset Participant in Past 30 Days	0.9	1.3	1.4	1.4	**-2.3***
Number of Housed Network Members	3.2	2.3	3.0	2.3	0.4
Number of Unhoused Network Members	1.6	1.7	2.1	1.6	**-2.5***

Significance values ^p≤ 0.10, *p ≤ 0.05, **p ≤ 0.01, ***p ≤0.001
